# Suppression of microRNA-222-3p ameliorates ulcerative colitis and colitis-associated colorectal cancer to protect against oxidative stress *via* targeting BRG1 to activate Nrf2/HO-1 signaling pathway

**DOI:** 10.3389/fimmu.2023.1089809

**Published:** 2023-01-27

**Authors:** Xue-jun Wang, Dan Zhang, Yan-ting Yang, Xiao-ying Li, Hong-na Li, Xiao-peng Zhang, Jun-yi Long, Yun-qiong Lu, Li Liu, Guang Yang, Jie Liu, Jue Hong, Huan-gan Wu, Xiao-peng Ma

**Affiliations:** ^1^ Yueyang Hospital of Integrated Traditional Chinese and Western Medicine, Shanghai University of Traditional Chinese Medicine, Shanghai, China; ^2^ Eye Institute and Department of Ophthalmology, Eye & ENT Hospital, Fudan University, Shanghai, China; ^3^ Shanghai Research Institute of Acupuncture and Meridian, Shanghai University of Traditional Chinese Medicine, Shanghai, China

**Keywords:** ulcerative colitis (UC), colitis-associated colorectal cancer (CAC), oxidative stress, miR-222-3p, BRG1/Nrf2/HO-1 pathway

## Abstract

Oxidative stress is an important pathogenic factor in ulcerative colitis (UC) and colitis-associated colorectal cancer (CAC), further impairing the entire colon. Intestinal epithelial cells (IECs) are crucial components of innate immunity and play an important role in maintaining intestinal barrier function. Recent studies have indicated that microRNA-222-3p (miR-222-3p) is increased in colon of UC and colorectal cancer (CRC) patients, and miR-222-3p is a crucial regulator of oxidative stress. However, whether miR-222-3p influences IEC oxidative stress in UC and CAC remains unknown. This study investigated the effect of miR-222-3p on the regulation of IEC oxidative stress in UC and CAC. An *in vitro* inflammation model was established in NCM460 colonic cells, mouse UC and CAC models were established *in vivo*, and IECs were isolated. The biological role and mechanism of miR-222-3p-mediated oxidative stress in UC and CAC were determined. We demonstrated that miR-222-3p expression was notably increased in dextran sulfate sodium (DSS)-induced NCM460 cells and IECs from UC and CAC mice. *In vitro*, these results showed that the downregulation of miR-222-3p reduced oxidative stress, caspase-3 activity, IL-1β and TNF-α in DSS-induced NCM460 cells. We further identified BRG1 as the target gene of miR-222-3p, and downregulating miR-222-3p alleviated DSS-induced oxidative injury *via* promoting BRG1-mediated activation Nrf2/HO-1 signaling in NCM460 cells. The *in vivo* results demonstrated that inhibiting miR-222-3p in IECs significantly relieved oxidative stress and inflammation in the damaged colons of UC and CAC mice, as evidenced by decreases in ROS, MDA, IL-1β and TNF-α levels and increases in GSH-Px levels. Our study further demonstrated that inhibiting miR-222-3p in IECs attenuated oxidative damage by targeting BRG1 to activate the Nrf2/HO-1 signaling. In summary, inhibiting miR-222-3p in IECs attenuates oxidative stress by targeting BRG1 to activate the Nrf2/HO-1 signaling, thereby reducing colonic inflammation and tumorigenesis.

## Introduction

Ulcerative colitis (UC), a type of inflammatory bowel disease (IBD), is a chronic inflammatory disease of the colonic mucosa. The peak age of UC onset is between 30 and 40 years, which is increasing in incidence and prevalence (1, 2). UC patients have significantly increased risks of developing colorectal cancer (CRC) in the long term, and chronic inflammation is a driver of tumor progression (3, 4). Studies have shown that the deficiency of intestinal epithelial cells(IECs) homeostasis maintenance can lead to chronic inflammation and inflammatory cancer transformation (5–8).

Oxidative stress has long been recognized as one of the main pathogenic factors of UC and colitis-associated colorectal cancer (CAC) (9). Under normal conditions, reactive oxygen species (ROS) in intestinal tissue have bactericidal effects and participate in intestinal defense. However, excessive ROS production exceeds the buffering capacity of the host’s antioxidant defense, and the resulting oxidative stress can lead to lipid peroxidation, inflammatory responses, and intestinal mucosal barrier damage (9, 10). ROS generated by chronic inflammatory infiltration are thought to contribute to the generation of dysplastic lesions. Excessive ROS production can lead to DNA damage, which can become oncogenic (11, 12).

MicroRNAs (miRNAs, miRs) are noncoding single-stranded small RNAs approximately 17-25 nucleotides in length that are critical regulators of gene expression (13). MiRNAs can bind to the 3’ untranslated region (3’-UTR) of mRNAs, resulting in translational inhibition or the degradation of target mRNAs (14). MiRNAs have been shown to play critical roles in managing molecular and cellular processes in cancer and inflammation (15, 16). Among miRNAs, microRNA-222-3p (miR-222-3p) is a major regulator of oxidative stress and inhibiting the expression of miR-222-3p can significantly reduce the development of oxidative stress (17, 18). Recently, there is evidence suggesting an increased in the miR-222-3p expression in the colonic mucosal tissues of UC patients and in the colorectal tissues of CRC patients (19, 20). However, whether miR-222-3p plays a role in regulating oxidative stress in the colon in UC and CAC has not been investigated.

Brahma-related gene 1 (BRG1; SMARCA4) is an ATPase subunit of the SWI/SNF chromatin remodeling complex, which changes the structure of chromatin by hydrolyzing the energy released by ATP (21, 22). Studies have shown that BRG1 is crucial to maintain the homeostasis of IECs to prevent colitis and tumorigenesis, and the expression of BRG1 in the IECs of UC patients is significantly reduced (12). BRG1 deletion led to excess ROS levels in mice, resulting in defective colonic barrier integrity, and the oxidative stress generated by ROS showed that the mice were highly susceptible to colitis and CAC (12, 23). Studies have shown that BRG1 is a potential target gene of miR-222-3p, and miR-222-3p directly binds to the 3’-UTR of BRG1, thereby inhibiting the transcription and translation of BRG1 (24). By inhibiting miR-222-3p, BRG1 expression can be significantly increased (24), and BRG1 further activates the antioxidant Nrf2/HO-1 signaling to resist oxidative stress (25, 26).

Nuclear factor erythroid 2-related factor 2 (Nrf2) is an critical transcription factor associated with the cellular antioxidant response and a central regulator that maintains intracellular redox homeostasis (27). Under physiological conditions, Nrf2 exists in the cytoplasm and combines with Kelch-like ECH-associated protein 1 (Keap1) to form a complex, and Nrf2 is degraded by proteases, maintaining an inhibited state (28–30). However, after exposure to oxidative stress, Nrf2 is released from the Keap1/Nrf2 complex and translocates into the nucleus to form a dimer with the small Maf protein, associates with the antioxidant response element (ARE) and further exerts antioxidant and anti-inflammatory effects by activating the downstream antioxidant protein heme oxygenase-1 (HO-1) (31).

**Figure 1:**
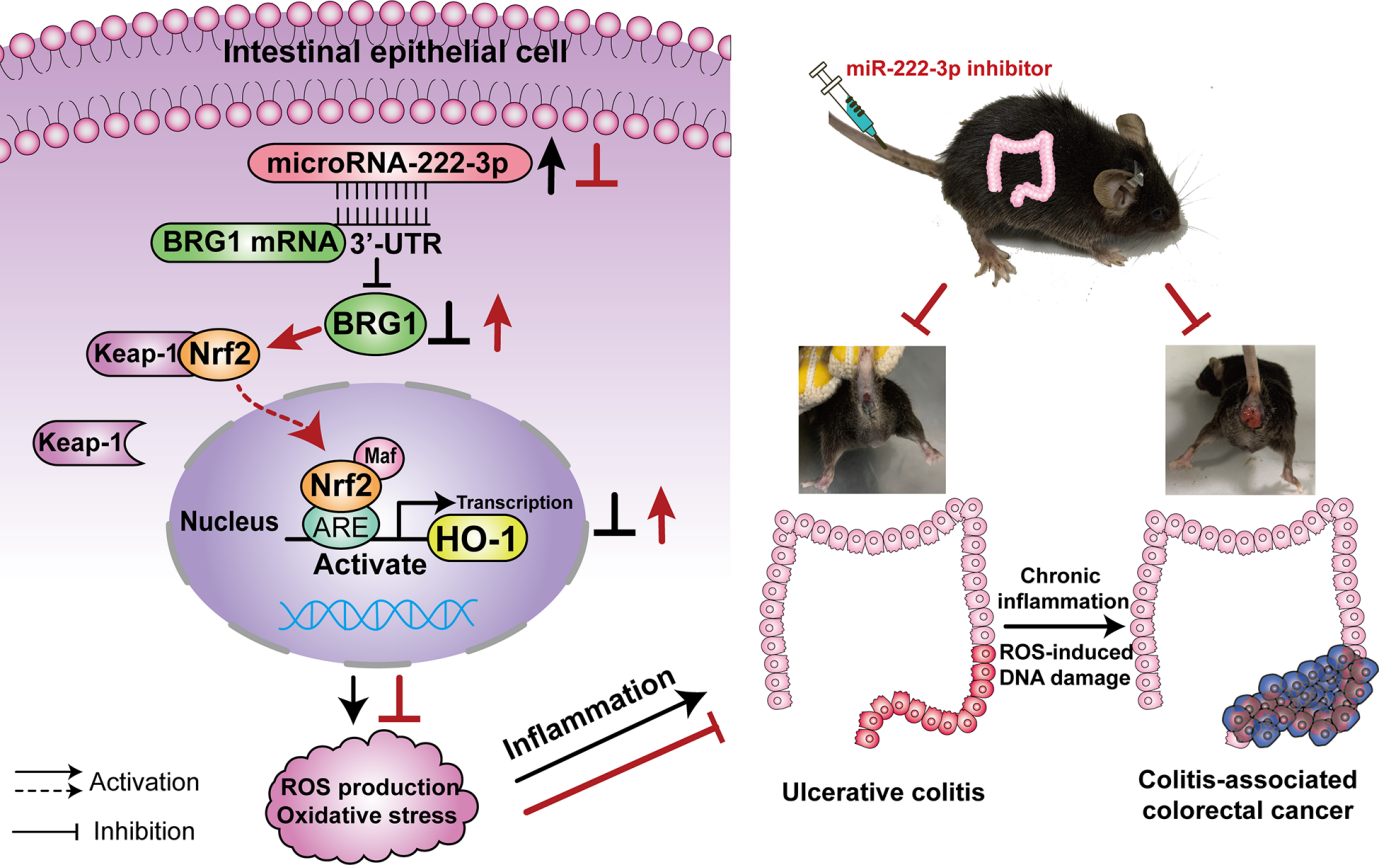
Illustration of the mechanisms of miR-222-3p in UC and CAC. MiR-222-3p directly controls oxidative stress *via* the BRG1/Nrf2/HO-1 pathway in IECs. MiR-222-3p overexpression leads to the inhibition of the BRG1/Nrf2/HO-1 pathway, which results in excessive ROS production and oxidative stress thereby compromising mucosal barrier integrity and promoting UC. Chronic inflammation combined with ROS-induced DNA damage promotes the malignant progression of CAC. UC, Ulcerative colitis; CAC, Colitis-associated colorectal cancer.

Therefore, we hypothesized that miR-222-3p could promote ulcerative colitis and tumorigenesis by regulating oxidative stress in IECs and that the BRG1/Nrf2/HO-1 pathway was involved in this process (**Figure 1**). Our study aims to explore the effect of miR-222-3p on the regulation of IECs oxidative stress in UC and CAC through the BRG1/Nrf2/HO-1 pathway.

## Materials and methods

### Cell culture

Human NCM460 colonic cells (INCELL, San Antonio, TX) were purchased from Ningbo Mingzhou Biotechnology Co., Ltd. (Ningbo, China) and cultured in Minimul Essential Medium (MEM, Gibco, Rockville, MD) supplemented with 10% (v/v) FBS and 1% penicillin/streptomycin (Gibco, Rockville, MD). The cells were cultured in a humidified atmosphere of 95% air and 5% CO_2_ at 37°C.

### Establishment of a dextran sulfate sodium-induced inflammation model in NCM460 cells

According to previous methods, NCM460 cells were placed under specific conditions (5% CO_2_/95% air) at 37°C for 24 h. Afterward, NCM460 cells were exposed to 20 mg/mL dextransulfate sodium (DSS) for 12 h (32, 33).

### Cell transfection

The miR-222-3p mimics, miR-222-3p inhibitor, and negative control miRNA (miR-NC) were purchased from Shanghai GenePharma (Shanghai, China) and transfected into cells using Lipofectamine 2000 (Thermo Fisher Scientific, USA) according to the manufacturer’s protocols. BRG1 shRNA and negative control shRNA (NC-shRNA) were purchased from Shanghai Genechem Co., Ltd. (Shanghai, China) and transfected into cells according to the manufacturer’s instructions.

### Cell counting kit-8 assay

NCM460 cell viability was assessed using a Cell Counting Kit-8 (CCK-8) assay from KeyGen BioTech (Nanjing, China) according to the manufacturer’s instructions. Briefly, the cells were added into 96-well plates at a density of 1×10^4^ cells/well and cultured for 24 h. After being transfected and treated, the cells were mixed with 10 μL/well CCK-8 reagent at 37°C for 1.5 h. NCM460 cell viability was determined by determining the absorbance at 450 nm using a microplate reader (BioTek, USA).

### Luciferase reporter assay

To validate BRG1 as the target gene of miR-222-3p, a dual-luciferase reporter assay was performed. Briefly, the mutated sequences of the binding sites were designed, and the corresponding vectors were constructed by Shanghai Genechem Co., Ltd. (Shanghai, China). The reporter vector was cotransfected with miR-222-3p mimics into NCM460 cells using Lipofectamine 2000 (Thermo Fisher Scientific, USA). After 48 h, according to the manufacturer’s instructions, the activity of luciferase was detected using a dual-luciferase reporter assay (Beyotime, Shanghai, China).

### RNA isolation and quantitative real-time PCR

The expression levels of miR-222-3p, BRG1, Nrf2 and HO-1 mRNA were measured by qRT−PCR. In brief, total RNA was extracted from NCM460 cells and mouse IECs with TRIzol reagent (Invitrogen, Carlsbad, CA, USA). For miRNA analysis, cDNA synthesis was carried out using an EZ-press microRNA Reverse Transcription Kit (EZBioscience, USA). For mRNA analysis, total RNA was reverse transcribed using the PrimeScript RT Reagent Kit with gDNA Eraser (Takara, Japan). qRT−PCR was performed with a Roche Light Cycler 480 II using TB Green™ Premix Ex Taq™ (Tli RNaseH Plus) (Takara, Japan). The relative amounts of transcripts were calculated using the 2^−ΔΔCt^ formula. To normalize the data, U6 served as the internal reference for miRNAs, and GAPDH as the internal reference for mRNAs. The specific primer sequences were shown in **Supplementary Table 2**.

### Western blot analysis

The protein expression levels of BRG1, Nu-Nrf2, HO-1, Keap-1 and caspase-3 were measured by Western blotting. Generally, total proteins were extracted from NCM460 cells and mouse IECs using RIPA buffer containing protease inhibitors (Roche, Basel, Switzerland), and nuclear proteins were extracted from cells with a nuclear protein extraction kit (Beyotime, Shanghai, China). Then, the protein concentrations were detected with a BCA protein assay kit (Beyotime, Shanghai, China). 10% SDS-PAGE was used to separate equal amount of proteins (40 μg) and transferred onto a PVDF membrane (Millipore, Boston, MA, USA) by electroblotting. The membrane was then blocked with 5% BSA and incubated with the appropriate primary antibodies, including anti-BRG1 (1:1000, Abcam, Cambridge, U.K), anti-Nrf2 (1:1000, CST, Danvers, MA, USA), anti-HO-1 (1:1000, CST, Danvers, MA, USA), anti-Keap-1 (1:1000, Abcam, Cambridge, U.K.), anti-β-actin (1:1000, Beyotime, Shanghai, China), and anti-Lamin B2 (1:1000, CST, Danvers, MA, USA) at 4°C overnight. Afterward, the membrane was incubated with secondary antibodies (1:5000; Beyotime, Shanghai, China) conjugated with peroxidase for 1 h at room temperature. The proteins were detected by an enhanced chemiluminescence (ECL) kit (Beyotime, Shanghai, China). Protein expression was analyzed using Image J software.

### Dichlorofluorescein diacetate assay

ROS generation was detected using a 6-carboxy-2′,7′-dichlorodihydrofluorescein diacetate (DCFH-DA) ROS assay kit (Beyotime, Shanghai, China). For NCM460 cells were treated with DCFH-DA (10 μmol/L) and incubated for 20 min at 37°C. Afterward, NCM60 cells were observed under a fluorescence microscope, and the IOD intensity of ROS was analyzed by Image-Pro Plus. For IECs, cells were treated with DCFH-DA (10 μmol/L) and incubated for 20 min at 37°C. The fluorescence intensity in IECs was detected using a fluorescence microplate reader (488 nm excitation wavelength and 525 nm emission wavelength).

### Glutathione peroxidase assay and malondialdehyde assay

According to the manufacturer’s protocol, glutathione peroxidase (GSH-Px) activities of NCM460 cells and mouse IECs were determined using a total glutathione peroxidase assay kit (Beyotime, Shanghai, China), and malondialdehyde (MDA) levels of NCM460 cells and mouse IECs were measured using a lipid peroxidation MDA assay kit (Beyotime, Shanghai, China).

### Caspase-3 activity assay

Apoptosis of NCM460 cells and mouse IECs were measured using a Caspase 3 Activity Assay Kit (Beyotime, Shanghai, China). Briefly, cells were lysed with cell lysis buffer, and the supernatant was collected by centrifugation. Then, 50 μL supernatant was added to 40 μL assay buffer and 10 μL caspase-3 substrate and incubated in 96-well plates (Ac-DEVD-pNA) at 37° C for 6 h. The absorbance values at 405 nm were analyzed by a microplate reader (BioTek, USA).

### Enzyme-linked immunosorbent assay

The levels of IL-1β and TNF-α in the cell-free supernatants of NCM460 cells and mouse IECs were examined by enzyme-linked immunosorbent assay (ELISA) using IL-1β (Shanghai Simuwu Biotechnology Co., Ltd, Shanghai, China) and TNF-α enzyme-linked immunosorbent assay kits (Shanghai Simuwu Biotechnology Co., Ltd, Shanghai, China), respectively. Cell-free supernatants were collected, and IL-1β and TNF-α levels were measured according to the manufacturer’s instructions. The absorbance at 450 nm was measured by a microplate reader (BioTek, USA).

### Mice ethics

Male C57BL/6 mice (6-8 weeks old) were purchased from Shanghai SLAC Laboratory Animal Co., Ltd. (Shanghai, China; License no: SYXK (Shanghai) 2018-0040) and used for DSS-induced UC models and azoxymethane (AOM)/DSS-induced CAC mouse models. All animal experiment protocols were implemented in accordance with the International Guiding Principles for Biomedical Research Involving Animals recommended by the World Health Organization and were approved by the Ethics Committee of Yueyang Clinical Medicine School, Shanghai University of Traditional Chinese Medicine (No. YYLAC-2020-094-1, YYLAC-2020-085-1).

### AAV9-GFP construction and tail vein injection

The miR-222-3p inhibitor (AAV-222-3p inhibitor) and a negative control (AAV-NC) recombinant adeno-associated virus-green fluorescence protein vector 9 (AAV9-GFP) was obtained from Shanghai Genechem Co., Ltd. (Shanghai, China). In brief, 1 × 10^11^ particles of AAV in 200 μL PBS were injected into the tail veins of mice. After 2 months, the colon and rectum were removed, and the fluorescence intensity of AAV9-GFP was measured with a fluorescence microscope (**Supplementary Figures 1**, **2**).

### Induction of UC and CAC

According to a previous study (12), AAVs were first injected into mice’s tail veins. Two months later, the mice were first subjected to BRG1 deletion with tamoxifen (100 mg/kg body weight) (Sigma−Aldrich, USA) for 3 consecutive days, and then the UC and CAC models were induced. To induce UC, the mice were fed 3% DSS (MW, 36–50 kDa; MP Biomedicals) for 3 days, followed by regular drinking water. In the CAC experiments, mice were intraperitoneally injected with AOM (10 mg/kg body weight) (Sigma−Aldrich, USA). After 7 days, 3% DSS was offered *via* drinking water for 4 days, followed by 16 days of normal drinking water. This DSS treatment cycle was repeated three times. The following day, the mice were sacrificed, and the large intestines were dissected to record the size and number of tumors. Intestinal tissues were then fixed in 4% paraformaldehyde for H&E staining.

### Assessment of colonic inflammation

The disease activity index (DAI) and colon macroscopic damage indices (CMDIs) were measured to evaluate the symptoms of UC in the mice. The DAI was measured according to a previous standard scoring system (34). Mouse body weight, colon length, stool consistency, and rectal bleeding were observed. Briefly, the DAI scores were defined as follows: weight loss (0 = no loss, 1 = 5–10%, 2 = 10–15%, 3 = 15–20%, 4=>20%); rectal bleeding (0= no blood, 1 = occult blood 1+, 2 = occult blood 2+, 3 = occult blood 3+, 4 = occult blood 4+); and the appearance of diarrhea (0 = none, 2 = mild diarrhea, 4 = gross diarrhea). The mice were sacrificed, the abdominal cavity was opened, and the length and weight of the colon were recorded. Subsequently, the colon tissues were washed in ice-cold PBS to clear fecal residue. CMDIs were assessed (35). The scores are shown in **Supplementary Table 1**.

### Isolation and culture of IECs

IECs were isolated from mice according to a previous study (12). The colons were cut into 6-8 mm pieces, rinsed three times with ice cold PBS containing 2% fetal calf serum, and incubated with digestion buffer (5% penicillin/streptomycin with 1% collagenase type I and 1% Dispase II) for 45 min at 37 °C. Then, the cells were passed through a 100 μm cell strainers (Corning, USA). After being centrifuged, the cells were plated in petri dishes overnight and cultured in epithelial cell culture medium with 1% penicillin/streptomycin and 10% Fetal bovine serum (FBS). The next day, the cells were digested with 0.25% trypsin (containing EDTA) for 2 min to remove other cells. Subsequently, the supernatant was removed, and 0.25% trypsin (containing EDTA) was added for 4 min for digestion. Finally, IECs in suspension were collected and placed in a petri dish for 24 h. The purity of the IECs was determined by immunofluorescence (IF) analysis (**Supplementary Figure 3**).

The isolated IECs were incubated for 24 h, after which cells were fixed with 4% paraformaldehyde, permeabilized in 0.25% Triton X-100 and incubated with 5% BSA. After being incubated with diluted antibodies against CK19 (1:100, Proteintech, USA) at 4°C overnight, the cells were exposed to secondary antibodies conjugated with the fluorophore FITC (1:500, Proteintech, USA) for 2 h in the dark at room temperature and treated with DAPI (1:1000, Beijing Solarbio Science & Technology Co., Ltd, Beijing, China) for 5 min. The cells were observed with a fluorescence microscope and photographed (Olympus Corporation, Japan).

### Hematoxylin and eosin staining

Histopathological specimens of colon tissues were fixed with 4% paraformaldehyde. The samples were embedded in paraffin and sliced into 4 μm sections. The prepared paraffin sections were stained with H&E (36), sealed and observed using a microscope (Olympus Corporation, Japan).

### Immunohistochemistry

Immunohistochemistry was performed to analyze the expression of BRG1 in the colon tissues according to standard protocols. The sections were heated at 60°C and deparaffinized. Then, the sections were placed in citric acid for antigen retrieval, and blocked with 5% BSA for 20 min, followed by stained with an anti-BRG1 antibody (1:200, Abcam, Cambridge, U. K) overnight at 4°C. The next day, the sections were observed with a microscope (Olympus Corporation, Japan).

### Statistical analysis

SPSS 25.0 statistical software (IBM, Armonk, NY, USA) was used for statistical analyses. All data (except for the CMDIs score) in the different experimental groups are presented as the mean ± SD and were analyzed using one-way ANOVA test. The least significant difference (LSD) method was used when pairwise tests indicated that the variances of the different groups were equal, and the Games-Howell method was used when the variances were unequal. CMDIs data are expressed as the median (*P25, P75*) and were analyzed using a nonparametric test (Kruskal−Wallis). *P* < 0.05 was considered statistically significant.

## Results

### miR-222-3p downregulation protects NCM460 cells from DSS-induced injury

**Figure 2:**
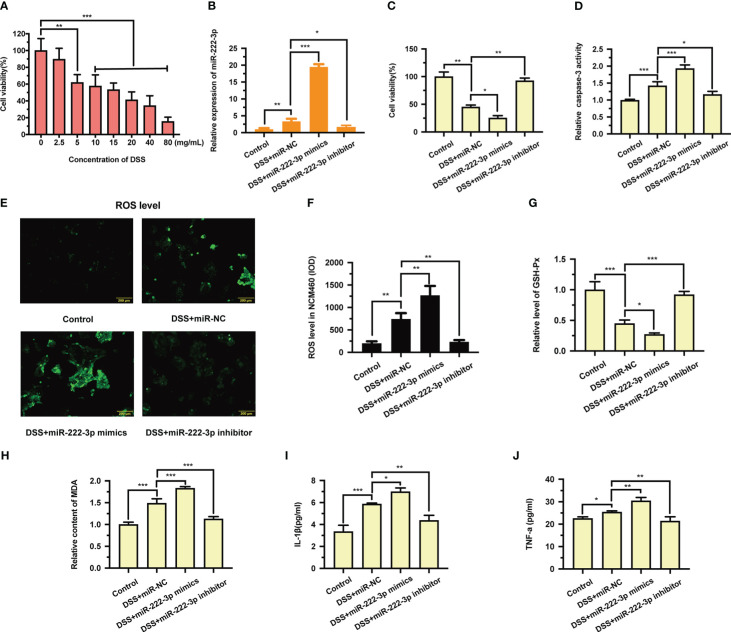
Effects of miR-222-3p on DSS-induced cell injury in the NCM460 cells. **(A)** Effect of DSS on NCM460 cell viability was detected by CCK-8 assay. **(B)** The effect of miR-222-3p mimics or inhibitor transfection on miR-222-3p expression was assessed by qRT-PCR from NCM460 cells. **(C)** Effect of miR-222-3p on NCM460 cell viability was measured using the CCK-8 assay. **(D)** Effect of miR-222-3p on NCM460 cell apoptosis was measured by caspase-3 activity assay. **(E, F)** Living cell microscopy. Effect of miR-222-3p on NCM460 cell ROS level was measured by DCFH-DA ROS assay kit. **(G, H)** Effect of miR-222-3p on the level of GSH-Px **(G)** and the content of MDA **(H)** were measured by corresponding kits from NCM460 cells. **(I, J)** Effect of miR-222-3p on the inflammatory factors, including IL-1β and TNF-α in the NCM460 cell supernatant were determined by ELISA. Data are presented as the mean ± SD (n = 3). ^*^ *P* < 0.05, ^**^ *P* < 0.01, ^***^ *P *< 0.001. Control: control group; DSS+miR-NC: DSS+negative control miRNA group; DSS+miR-222-3p mimics: miR-222-3p mimics group; DSS+miR-222-3p inhibitor: miR-222-3p inhibitor group. Scale bar: 200 *µ*m.

DSS has been reported to induce injury in cell and animal models of UC (33). To establish a UC model at the cellular level, a DSS-induced NCM460 cell injury model was constructed in our study. We first used the CCK-8 assay to evaluate the damage to DSS-induced NCM460 cells. As shown in **Figure 2A**, various concentrations of DSS decreased cell viability. In particular, 20 mg/mL DSS reduced the viability of NCM460 to less than 50%. To understand the biological function of miR-222-3p in regulating NCM460 cell injury under inflammatory conditions, we conducted miR-222-3p gain-of-function and loss-of-function experiments. We found that miR-222-3p expression was significantly higher in cells treated with DSS (**Figure 2B**), and the transfection of miR-222-3p mimics further significantly promoted the expression of miR-222-3p transfection of a miR-222-3p inhibitor markedly downregulated the expression of miR-222-3p in cells compared with transfection of a negative control (**Figure 2B**). The results suggest that miR-222-3p is a DSS-responsive miRNA in NCM460 cells.

We then examined the effect of miR-222-3p on cell viability by CCK-8 assay. We found that DSS significantly reduced cell viability, while miR-222-3p overexpression further decreased the viability of DSS-induced NCM460 cells (**Figure 2C**). However, miR-222-3p downregulation significantly increased DSS-induced NCM460 cell viability (**Figure 2C**). Caspase-3 activity showed that miR-222-3p overexpression significantly promoted DSS-induced apoptosis, whereas miR-222-3p downregulation decreased DSS-induced apoptosis (**Figure 2D**). These results suggest that inhibiting miR-222-3p protects NCM460 cells from DSS-induced cell viability impairment and apoptosis.

To further explore the biological function of miR-222-3p in DSS-induced NCM460 cell injury, we measured the effect of miR-222-3p on DSS-induced oxidative stress and inflammation. We found that miR-222-3p overexpression promoted ROS and MDA generation in DSS-induced NCM460 cells (**Figures 2E, F, H**), while inhibiting miR-222-3p significantly reduced the production of ROS and MDA in DSS-induced cells (**Figures 2E, F, H**). In addition, miR-222-3p overexpression decreased the level of glutathione peroxidase (GSH-Px) in DSS-induced NCM460 cells, while miR-222-3p knockdown enhanced it (**Figure 2G**).

We examined inflammatory factors in DSS-treated NCM460 cell supernatants and found that IL-1β and TNF-α were increased, and miR-222-3p overexpression further upregulated IL-1β and TNF-α levels in DSS-induced NCM460 cell supernatants (**Figures 2I, J**). When DSS-induced NCM460 cells were treated with the miR-222-3p inhibitor, IL-1β and TNF-α were significantly decreased (**Figures 2I, J**). These results show that miR-222-3p downregulation reduced oxidative stress and inflammation in DSS-induced NCM460 cells.

### BRG1 is a potential target gene of miR-222-3p

To explore the underlying mechanism of miR-222-3p regulating NCM460 cell injury under inflammatory condition, we searched for potential downstream target genes of miR-222-3p by bioinformatic analysis. Studies have shown that BRG1 is a key regulator of oxidative stress and apoptosis (12, 23) and is predicted to be a target gene of miR-222-3p (24).

**Figure 3:**
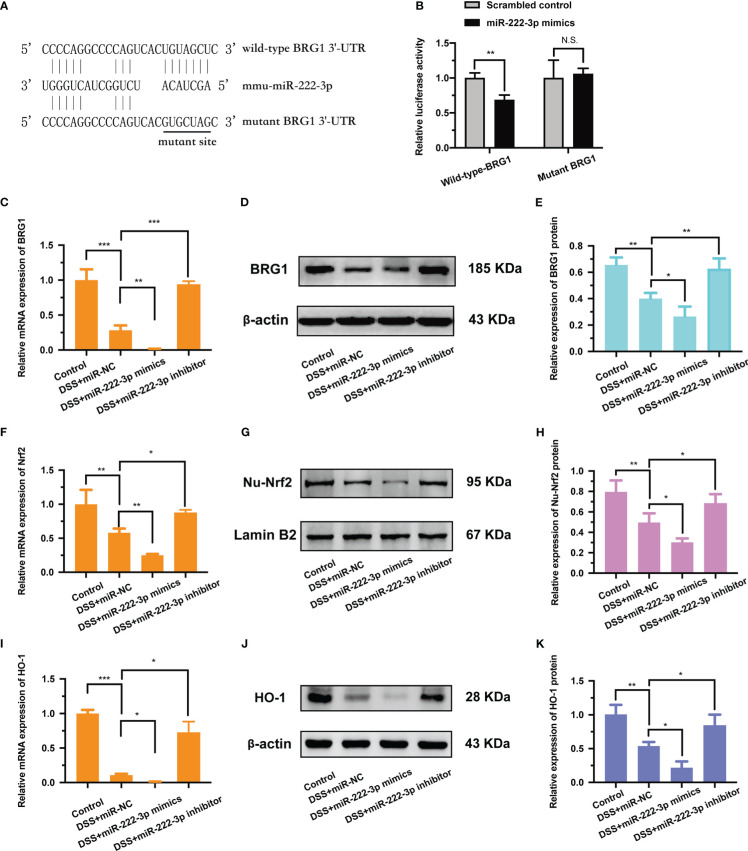
BRG1 is the potential target gene of miR-222-3p and inhibition of miR-222-3p promotes the activation of Nrf2/HO-1 signaling. **(A)** The alignment of miR-222-3p with the seed regions in BRG1 3′-UTR. **(B)** Designed wild-type or mutant sequences of BRG1 binding to miR-222-3p, and relative luciferase activity was detected by dual-luciferase reporter assay. **(C)** Relative BRG1 mRNA expression was determined by qRT-PCR from NCM460 cells. **(D, E)** Relative BRG1 protein expression was determined by Western blot from NCM460 cells. **(F)** Relative Nrf2 mRNA expression was determined by qRT-PCR from NCM460 cells. **(G, H)** Relative Nu-Nrf2 protein expression was determined by Western blot from NCM460 cells. **(I)** Relative HO-1 mRNA expression was determined by qRT-PCR from NCM460 cells. **(J, K)** Relative HO-1 protein expression was determined by Western blot from NCM460 cells. Data are presented as the mean ± SD (n = 3). ^*^ *P* < 0.05, ^**^ *P* < 0.01, ^***^ *P *< 0.001. Control: control group; DSS+miR-NC: DSS+negative control miRNA group; DSS+miR-222-3p mimics: miR-222-3p mimics group; DSS+miR-222-3p inhibitor: miR-222-3p inhibitor group. Scale bar: 200 *µ*m.

The 3′-UTR of BRG1 has a conserved binding site for miR-222-3p (**Figure 3A**). To investigate whether miR-222-3p directly targets to the BRG1 3′-UTR, a dual luciferase assay was performed (**Figure 3B**). The results showed that miR-222-3p overexpression significantly inhibited the luciferase activity of the luciferase reporter vector containing the wild-type BRG1 3′-UTR (**Figure 3B**). However, miR-222-3p overexpression did not have any effect on the luciferase activity of the reporter vector containing the mutant BRG1 3′-UTR (**Figure 3B**). Therefore, we found that miR-222-3p directly bound to BRG1.

In addition, we also detected the regulatory effect of miR-222-3p on BRG1. The results showed that BRG1 expression was inhibited by miR-222-3p overexpression, while promoted by miR-222-3p inhibition (**Figures 3C, D, E**). Overall, these results demonstrate that miR-222-3p can directly bind to BRG1 and negatively regulate its expression.

### Inhibiting miR-222-3p promotes the activation of Nrf2/HO-1 signaling

Studies have shown that BRG1 alleviates oxidative stress by activating Nrf2/HO-1 signaling (25, 26). Considering the regulation of BRG1 by miR-222-3p, we supposed that miR-222-3p could regulate the Nrf2/HO-1 signaling pathway. Our results demonstrated that miR-222-3p overexpression impaired nuclear Nrf2 protein expression and intracellular Nrf2 mRNA expression (**Figures 3F-H**), while miR-222-3p inhibition markedly promoted nuclear Nrf2 protein expression and intracellular Nrf2 mRNA expression (**Figures 3F-H**).

Moreover, miR-222-3p overexpression downregulated the mRNA (**Figure 3I**) and protein (**Figures 3J, K**) expression of HO-1 in DSS-induced NCM460 cells, whereas miR-222-3p inhibition upregulated the mRNA (**Figures 3I**) and protein (**Figures 3J, K**) expression of HO-1. These results suggest that miR-222-3p downregulation promotes the activation of Nrf2/HO-1 signaling.

### Downregulation of BRG1 reverses the protective effect of miR-222-3p inhibition on DSS-induced NCM460 cell injury

**Figure 4:**
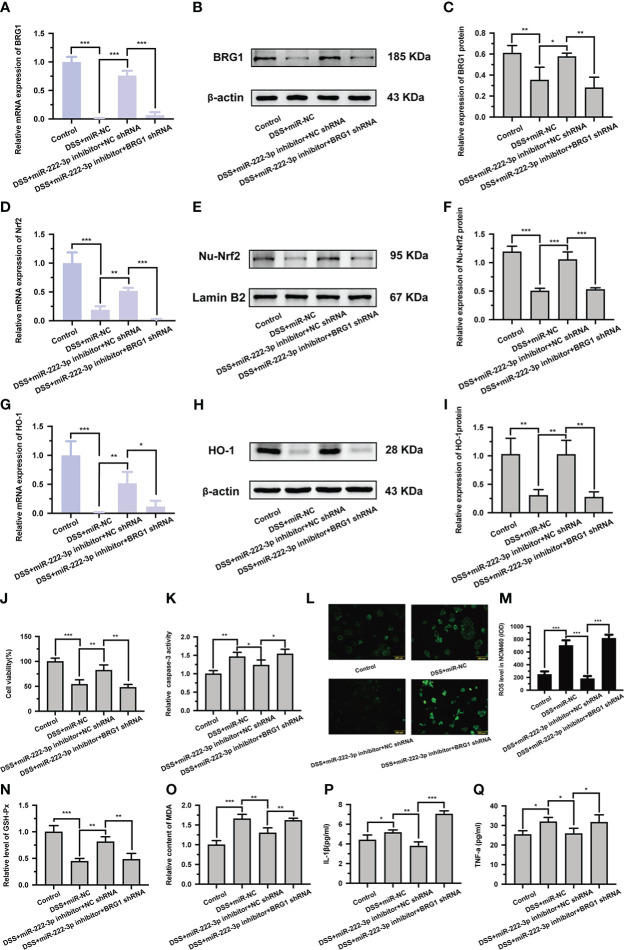
Knockdown of BRG1 reverses the protective effect of miR-222-3p inhibition in DSS-induced NCM460 cell injury. **(A)** Relative BRG1 mRNA expression was determined by qRT-PCR from NCM460 cells. **(B, C)** Relative BRG1 protein expression was determined by Western blot from NCM460 cells. **(D)** Relative Nrf2 mRNA expression was determined by qRT-PCR from NCM460 cells. **(E, F)** Relative Nu-Nrf2 protein expression was determined by Western blot from NCM460 cells. **(G)** Relative HO-1 mRNA expression was determined by qRT-PCR from NCM460 cells. **(H, I)** Relative HO-1 protein expression was determined by Western blot from NCM460 cells. **(J)** NCM460 cell viability was assessed using the CCK-8 assay. **(K)** NCM460 cell apoptosis was measured by caspase-3 activity assay. **(L, M)** Living cell microscopy. NCM460 cell ROS level was measured by DCFH-DA ROS assay kit. **(N, O)** the level of GSH-Px **(N)** and the content of MDA **(O)** were measured by corresponding kits from NCM460 cells. **(P, Q)** The inflammatory factors of IL-1β and TNF-α in the cell supernatant were determined by ELISA from NCM460 cells. Data are presented as the mean ± SD (n = 3). ^*^ *P* < 0.05, ^**^ *P* < 0.01, ^***^ *P *< 0.001. Control: control group; DSS+miR-NC: DSS+negative control miRNA group; DSS+miR-222-3p inhibitor+NC shRNA: miR-222-3p inhibitor+ negative control shRNA group; DSS+miR-222-3p inhibitor+BRG1 shRNA: miR-222-3p inhibitor+BRG1 shRNA group. Scale bar: 200 *µ*m.

To further confirm whether miR-222-3p inhibition relieves DSS-induced NCM460 cell injury by upregulating BRG1 expression, we examined the effect of BRG1 downregulation on the protection induced by miR-222-3p inhibition. We found that the transfection of BRG1 shRNA significantly abrogated the promotion of BRG1 expression induced by miR-222-3p inhibition (**Figures 4A-C**). Moreover, the promotion of Nrf2/HO-1 signaling by miR-222-3p inhibition was also significantly abolished by BRG1 knockdown (**Figures 4D-I**). As expected, the protective effect of miR-222-3p inhibition against DSS-induced NCM460 cell necrosis, apoptosis, oxidative stress and inflammation was also reversed by BRG1 knockdown (**Figures 4J-Q**). Overall, these results demonstrated that miR-222-3p downregulation attenuates DSS-induced NCM460 cell injury by upregulating BRG1 expression.

### The miR-222-3p inhibitor relieves DSS-induced colonic injury

Considering the impact of miR-222-3p on UC, we hypothesized that the miR-222-3p inhibitor would have therapeutic potential and tested the effect of the miR-222-3p inhibitor *in vivo* using a DSS-induced UC mouse model. Recombinant adeno-associated virus (AAV) vector-mediated gene transfer to IECs provides a mature method of intestinal transduction (37). Based on previous studies, we selected AAV serotype 9 with high intestinal transduction efficiency (38), and transfected mice with AAV-miR-222-3p inhibitor (AAV-222-3p inhibitor) or AAV-negative control vectors (AAV-NC) through tail vein injection. To determine intestinal efficiency *in vivo*, frozen colon tissue sections were analyzed by fluorescence to measure the scatteration and expression of the AAV-222-3p inhibitor and AAV-NC in the mice. We showed that the AAV-222-3p inhibitor and AAV-NC were dramatically present in colonic epithelial cells (**Supplementary Figure 1**).

**Figure 5:**
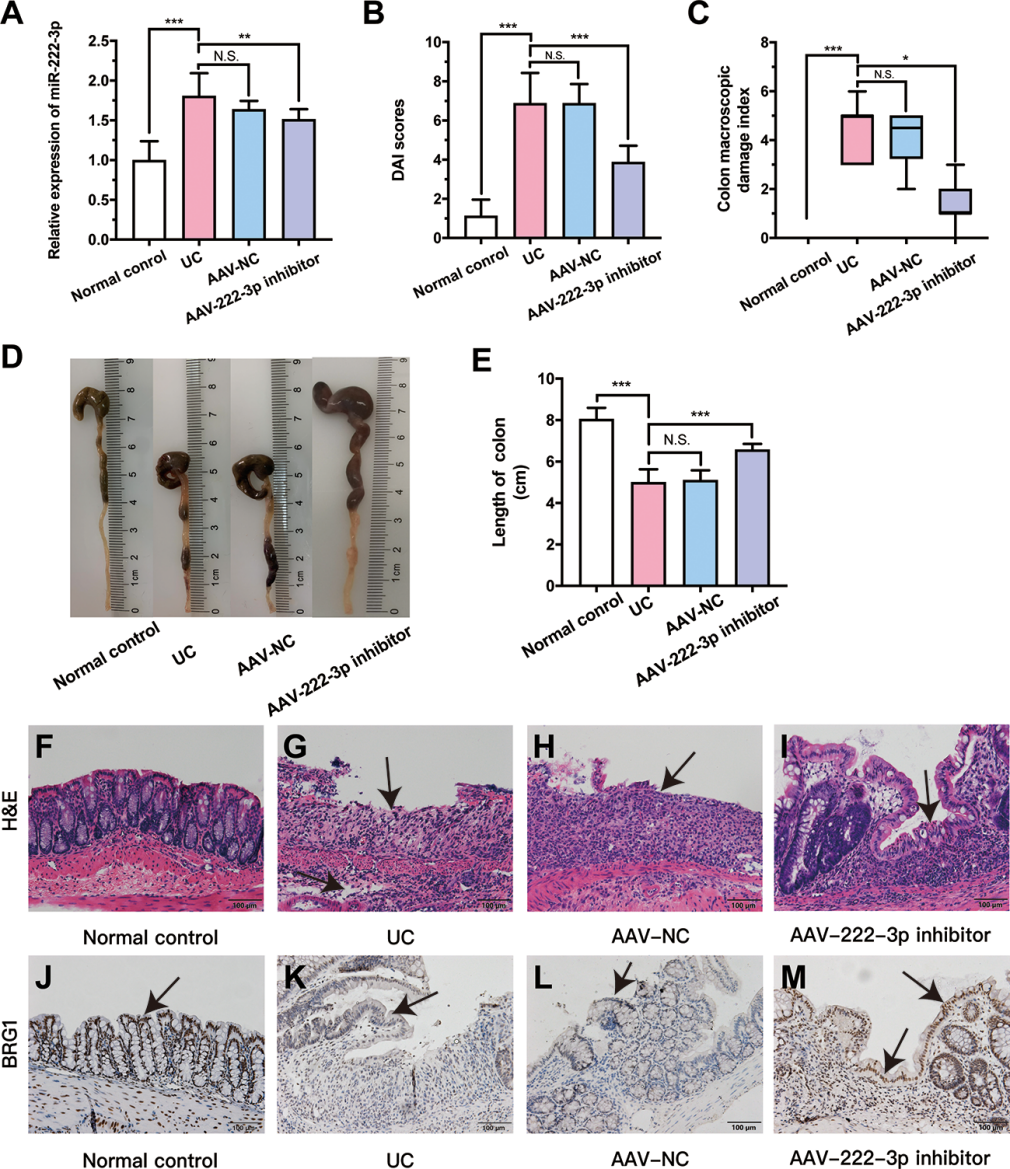
MiR-222-3p inhibitor relieves DSS-induced damage of colon. **(A)** qRT-PCR analysis of miR-222-3p in the IECs from DSS-induced mice. **(B)** DAI scores. **(C)** CMDIs. **(D)** Colon images. **(E)** Colon length. (F~I) morphological observation with hematoxylin and eosin (H&E)-stained colon sections as indicated, and arrows indicate ulcer healing sites. (J~M) Immunohistochemical analyses of BRG1 expression in colon tissues, and arrows indicate IECs locations. Data are presented as the median (*P25, P75*) (n = 8) in **(C)** and mean ± SD in **(A, B, E)** (n = 8). ^*^ *P* < 0.05, ^**^ *P* < 0.01, ^***^ *P *< 0.001. Normal control: Normal control group; UC: UC group; AAV-NC: UC+ AAV-negative control group; AAV-222-3p inhibitor: UC+ miR-222-3p inhibitor group. N.S., no significance. Scale bar: 100 µm

After successful AAV transfection, we extracted mouse IECs. To identify cells extracted from the colon as IECs, we stained the cells with immunofluorescence. The results showed that after the cells were fluorescently stained with the IEC-specific marker CK19, they showed the unique spindle shape of IECs (**Supplementary Figure 2**). Therefore, the extracted cells were mouse IECs. To confirm the expression of miR-222-3p, we performed qRT−PCR. Compared with that in the normal control group, the miR-222-3p expression in the UC group was significantly increased. However, the miR-222-3p expression was significantly suppressed by transfection of miR-222-3p inhibitor (**Figure 5A**). These results further demonstrated that the miR-222-3p inhibitor was transfected into IECs.

Mice in the UC and AAV-NC groups exhibited significant appetite and weight loss, diarrhea, mucus bloody stool and colon injury, and these mice had higher DAI scores and CMDIs than normal control mice (**Figures 5B, C**). The miR-222-3p inhibitor ameliorated DSS-induced colitis manifestations, DAI scores and CMDIs (**Figures 5B, C**). The shortening of the colon can show the severity of colon injury. Compared with that in the normal control group, there was a decrease in colon length in the UC group (**Figures 5D, E**). In contrast, the colons of mice in the AAV-222-3p inhibitor group were considerably longer (**Figures 5D, E**).

Colonic pathophysiologic structure was observed by HE staining (**Figures 5F-I**). Intestinal epithelial necrosis, goblet cell disorder and loss, continuous ulcers, and a large number of inflammatory cells infiltration in the mucosa and submucosa were observed in the UC and AAV-NC groups (**Figures 5G, H**). Pretreatment with the miR-222-3p inhibitor significantly attenuated colonic damage. Damage to intestinal epithelial tissue, goblet cells and glands was repaired, and arrows indicate ulcer healing sites (**Figure 5I**). Thus, our results show that miR-222-3p is involved in colon inflammation and that a miR-222-3p inhibitor that can reach IECs can attenuate colonic immune inflammation in UC mice.

### The miR-222-3p inhibitor activates Nrf2/HO-1 signaling pathway in UC mouse IECs by targeting BRG1

**Figure 6:**
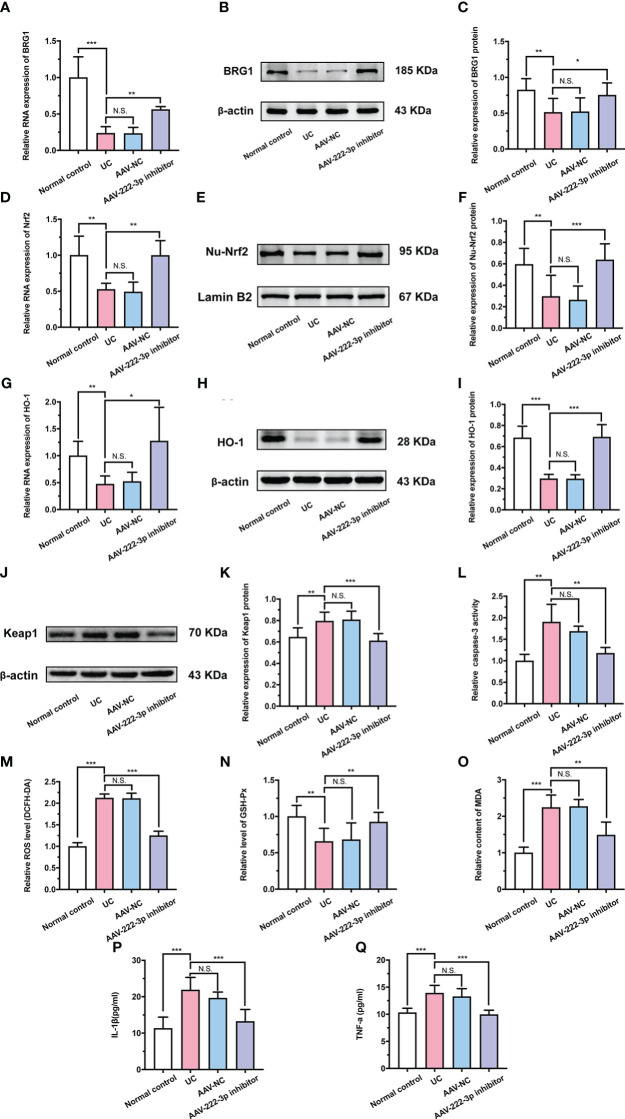
MiR-222-3p inhibitor attenuates oxidative damage by targeting BRG1 to activate Nrf2/HO-1 signaling pathway in IECs of UC mice. **(A)** Relative BRG1 mRNA expression was determined by qRT-PCR in the IECs from DSS-induced mice. **(B, C)** Relative BRG1 protein expression was determined by Western blot in the IECs from DSS-induced mice. **(D)** Relative Nrf2 mRNA expression was determined by qRT-PCR in the IECs from DSS-induced mice. **(E, F)** Relative Nu-Nrf2 protein expression was determined by Western blot in the IECs from DSS-induced mice. **(G)** Relative HO-1 mRNA expression was determined by qRT-PCR in the IECs from DSS-induced mice. **(H, I)** Relative HO-1 protein expression was determined by Western blot in the IECs from DSS-induced mice. **(J, K)** Relative Keap-1 protein expression was determined by Western blot in the IECs from DSS-induced mice. **(L)** IECs apoptosis was measured by caspase-3 activity assay from DSS-induced mice. **(M)** IECs ROS level was measured by DCFH-DA ROS assay kit from DSS-induced mice. **(N, O)** the level of GSH-Px **(N)** and the content of MDA **(O)** were measured by corresponding kits in the IECs from DSS-induced mice. **(P, Q)** IL-1β and TNF-α in the IECs supernatant were determined by ELISA from DSS-induced mice. Data are presented as the mean ± SD (n = 8). ^*^ *P* < 0.05, ^**^ *P* < 0.01, ^***^ *P *< 0.001. Normal control: Normal control group; UC: UC group; AAV-NC: UC+ AAV-negative control group; AAV-222-3p inhibitor: UC+ miR-222-3p inhibitor group. N.S., no significance.

As stated previously, the miR-222-3p inhibitor significantly decreased the level of miR-222-3p compared with that in the UC group. To explore the relationship between BRG1 and miR-222-3p in IECs of UC mice, we analyzed the BRG1 expression in the colon tissues of UC mice by immunohistochemistry (**Figure 5J-M**). The results revealed that there was lower BRG1 staining detected in IECs in the UC group than in the normal control group, while miR-222-3p inhibitor increased positive BRG1 staining in IECs (**Figures 5J-M**). In addition, we further detected the mRNA and protein expression of BRG1 in IECs by qRT−PCR and Western blotting (**Figures 6A-C**). The results also showed that the expression of BRG1 was decreased in the UC group compared with the normal control group. However, the miR-222-3p inhibitor improved BRG1 expression compared with that in the UC group. The changes in miR-222-3p and BRG1 expression in the DSS+miR-NC group and DSS+miR-222-3p inhibitor group were consistent with the *in vitro* results. The animal results were consistent with the cell experiments. Altogether, these results indicate that the expression levels of miR-222-3p and BRG1 are negatively correlated in the IECs of DSS-induced UC mice.

Research in recent years has revealed that BRG1 protects cells from oxidative damage by activating Nrf2/HO-1 signaling (39). Therefore, we further examined the relationship between BRG1 and Nrf2/HO-1 signaling in IECs. qRT−PCR and Western blotting were used to measure the protein and mRNA levels of Nrf2, HO-1 and Keap-1 (**Figures 6D-K**). As expected, the results showed that the miR-222-3p inhibitor increased the level of Nrf2 nuclear translocation and HO-1 in the IECs of DSS-induced UC mice (**Figures 6D-I**). Moreover, Western blot analysis showed that Keap-1 expression in the IECs of DSS-induced UC mice was apparently decreased by the miR-222-3p inhibitor (**Figures 6J, K**). These results showed that the miR-222-3p inhibitor activated Nrf2/HO-1 signaling in DSS-induced UC mouse IECs by targeting BRG1.

### The miR-222-3p inhibitor relieves oxidative stress and inflammation in IECs of UC mice

We further explored the effect of the miR-222-3p inhibitor on oxidative stress and inflammation in UC mice. As shown in **Figure 6L** and **Supplementary Figure 3**, DSS treatment induced marked apoptosis in IECs, while the miR-222-3p inhibitor significantly attenuated these changes. In addition, ROS and MDA levels in IECs were distinctly increased after DSS treatment (**Figures 6M, O**), while the miR-222-3p inhibitor markedly decreased ROS and MDA levels in the IECs of DSS-induced mice (**Figures 6M, O**). DSS treatment similarly decreased the GSH-Px level in IECs, while the miR-222-3p inhibitor reversed this change (**Figure 6N**). These results showed that the miR-222-3p inhibitor relieved apoptosis and oxidative stress in IECs from DSS-induced UC mice.

Then, the production of inflammatory cytokines was determined to evaluate the anti-inflammatory effects of the miR-222-3p inhibitor. As shown in **Figures 6P** and **Q**, DSS increased the levels of IL-1β and TNF-α in the supernatant of IECs, while the miR-222-3p inhibitor significantly inhibited these increases (**Figures 6P, Q**). These results demonstrated the efficacy of the miR-222-3p inhibitor in alleviating oxidative stress and inflammation in UC mice. In conclusion, the miR-222-3p inhibitor attenuates DSS-induced oxidative stress in IECs by targeting BRG1 to activate the Nrf2/HO-1 signaling pathway and thereby relieve inflammation.

### miR-222-3p inhibition in IECs protects mice from CAC

**Figure 7:**
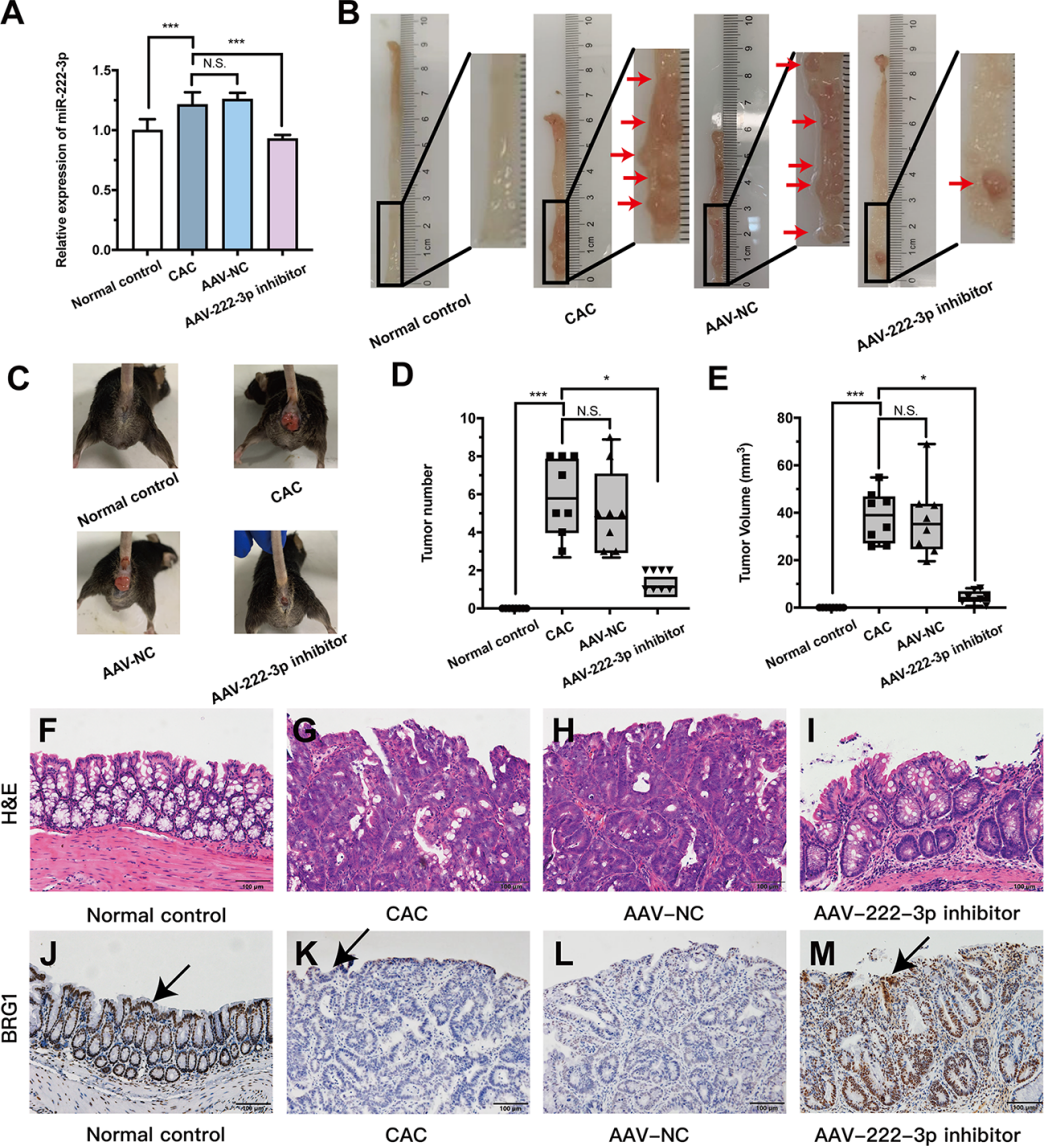
MiR-222-3p inhibitor plays CAC-suppressive roles *in vivo*. **(A)** qRT-PCR analysis of miR-222-3p in the IECs from AOM/DSS-induced mice. **(B)** Macroscopic images of tumors in the intestines of AOM/DSS-induced mice. **(C)** Images of AOM/DSS-induced mice. **(D, E)** Intestinal tumor numbers and tumor volumes were recorded. **(F–I)** morphological observation with H&E-stained colon sections as indicated. **(J–M)** Immunohistochemical analyses of BRG1 expression in colon tissues, and arrows indicate IECs locations. Data are presented as the median (*P25, P75*) (n = 8) in **(D, E)** and mean ± SD in **(A)** (n = 8). ^*^ *P* < 0.05, ^***^ *P *< 0.001. Normal control: Normal control group; CAC: CAC group; AAV-NC: CAC+ AAV-negative control group; AAV-222-3p inhibitor: CAC+ miR-222-3p inhibitor group. Scale bar: 100 µm. N.S., no significance.

To further determine whether miR-222-3p in IECs regulates the development of intestinal tumorigenesis, we used AOM/DSS to induce CAC in miR-222-3p inhibitor-treated mice. After successful AAV transfection (**Supplementary Figure 4**), we extracted mouse IECs. To confirm the expression of miR-222-3p, we performed qRT−PCR (**Figure 7A**). Consistent with the results in UC mice, the expression of miR-222-3p was also significantly elevated in the CAC group compared with the normal control group (**Figure 7A**). However, the miR-222-3p inhibitor decreased miR-222-3p expression (**Figure 7A**). These results further demonstrated that the miR-222-3p inhibitor was transfected into IECs.

Mice with CAC developed more and larger tumors than normal controls (**Figures 7B-E**). However, the numbers and volumes of macroscopically visible tumors were significantly decreased in miR-222-3p inhibitor mice compared with CAC mice (**Figures 7B-E**). Histologic analysis revealed that tumor cells could break through the basement membrane and grow invasively outward, and the nuclei become enlarged and hyperchromatic, reaching the level of adenocarcinoma in the CAC and AAV-NC groups (**Figures 7G, H**). In sharp contrast, the miR-222-3p inhibitor largely curtailed tumor development, the lesions that developed were mainly graded as mild-to-moderate dysplasia, and slightly enlarged and hyperchromatic nuclei were observed (**Figure 7I**). These results showed that the miR-222-3p inhibitor rendered the mice resistant to AOM/DSS-induced CAC.

### The miR-222-3p inhibitor protects IECs from oxidative stress by activating the BRG1/Nrf2/HO-1 pathway in CAC mice

**Figure 8:**
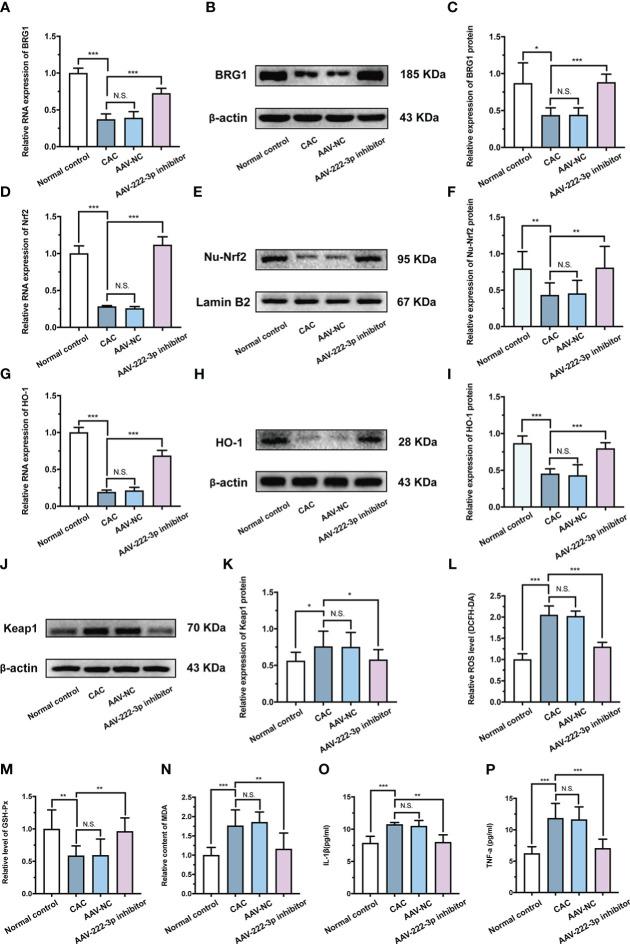
miR-222-3p inhibitor protects IECs from oxidative stress by activating the BRG1/Nrf2/HO-1 pathway in CAC mice. **(A)** Relative BRG1 mRNA expression was determined by qRT-PCR in the IECs from AOM/DSS-induced mice. **(B, C)** Relative BRG1 protein expression was determined by Western blot in the IECs from AOM/DSS-induced mice. **(D)** Relative Nrf2 mRNA expression was determined by qRT-PCR in the IECs from AOM/DSS-induced mice. **(E, F)** Relative Nu-Nrf2 protein expression was determined by Western blot in the IECs from AOM/DSS-induced mice. **(G)** Relative HO-1 mRNA expression was determined by qRT-PCR in the IECs from AOM/DSS-induced mice. **(H, I)** Relative HO-1 protein expression was determined by Western blot in the IECs from AOM/DSS-induced mice. **(J, K)** Relative Keap-1 protein expression was determined by Western blot in the IECs from AOM/DSS-induced mice. **(L)** IECs ROS level was measured by DCFH-DA ROS assay kit from AOM/DSS-induced mice. **(M, N)** the level of GSH-Px **(M)** and the content of MDA **(N)** were measured by corresponding kits in the IECs from AOM/DSS-induced mice. **(O, P)** IL-1β and TNF-α in the IECs supernatant were determined by ELISA from AOM/DSS-induced mice. Data are presented as the mean ± SD (n = 8). ^*^ *P* < 0.05, ^**^ *P* < 0.01, ^***^ *P *< 0.001. Normal control: Normal control group; CAC: CAC group; AAV-NC: CAC+ AAV-negative control group; AAV-222-3p inhibitor: CAC+ miR-222-3p inhibitor group. N.S., no significance.

Based on the UC results, we further explored the connection between BRG1 and miR-222-3p in AOM/DSS-induced colonic IECs. Immunohistochemistry showed that the BRG1 staining detected in IECs was also lower in AOM/DSS-induced mice than that in normal control group (**Figures 7J-M**). However, miR-222-3p inhibitor increased the positive BRG1 staining in IECs (**Figures 7J-M**). We further detected the mRNA and protein expression of BRG1. As shown in **Figures 8A-C**, compared to that in the normal control group, BRG1 expression was reduced in the IECs of AOM/DSS-induced mice, while miR-222-3p increased BRG1 expression. This result is consistent with the cell experiments and UC results. In conclusion, miR-222-3p can negatively regulate the expression level of BRG1 in DSS-induced or AOM/DSS-induced mouse IECs.

In addition, the results showed that the level of HO-1 and Nrf2 nuclear translocation in IECs were upregulated by miR-222-3p inhibitor treatment of AOM/DSS-induced CAC mice (**Figures 8D-I**). Moreover, the miR-222-3p inhibitor decreased the expression of Keap-1 (**Figures 8J, K**). These results showed that the miR-222-3p inhibitor activated Nrf2/HO-1 signaling in AOM/DSS-induced CAC mouse IECs by targeting BRG1.

We further explored the effect of the miR-222-3p inhibitor on oxidative stress and inflammation in AOM/DSS-induced colonic IECs. ROS and MDA levels in IECs were increased after AOM/DSS treatment (**Figures 8L, N**), while the miR-222-3p inhibitor markedly reversed these changes (**Figures 8L, N**). AOM/DSS treatment decreased GSH-Px levels in IECs, while the miR-222-3p inhibitor increased GSH-Px levels (**Figure 8M**). As shown in **Figures 8O** and **P**, DSS treatment increased the relative levels of IL-1β and TNF-α in the supernatants of IECs, while the miR-222-3p inhibitor significantly inhibited these changes (**Figures 8O, P**).

Collectively, these data indicated that the miR-222-3p inhibitor protected IECs from oxidative stress by activating the BRG1/Nrf2/HO-1 pathway in AOM/DSS-induced CAC mice, thereby inhibiting the inflammatory-cancer transition.

## Discussion

Although much attention has been given to the important role of adaptive immunity in intestinal mucosal inflammation, IECs are one of the most crucial components of innate immunity and play an important role in maintaining intestinal barrier function (40–42). A compromised epithelial barrier and concomitant hyperimmune response to the gut microbiota are implicated in the pathogenesis of IBD and CAC (12, 43, 44). Studies have reported that miRNAs are key regulators of IEC barriers, regulating the growth and apoptosis of IECs and tight junctions between IECs (13, 45–47). There is evidence to indicate that miR-222-3p expression is upregulated in the colonic mucosal tissues of UC patients and in the colorectal tissues of CRC patients (19, 20). MiR-222-3p inhibits VDR and activates STAT3 signaling pathway in RAW264.7 cells by targeting SOCS1, which in turn exacerbates inflammatory responses (19). Oxidative stress has long been recognized as one of the main pathogenic factors of UC and CAC (9, 48). MiR-222-3p is an important regulator of oxidative stress (18). Under oxidative stress conditions, the expression of miR-222-3p is significantly increased, and inhibiting miR-222-3p can significantly reduce oxidative stress and improve cellular oxidative damage and apoptosis (18, 49, 50). However, whether miR-222-3p is involved in the occurrence and development of UC and CAC by regulating oxidative stress in IECs is still unknown.

In our study, we demonstrated that IEC expression of miR-222-3p was significantly increased in mice with UC and CAC. We generated DSS-induced or AOM/DSS-induced mice to establish experimental models of UC and CAC and found that miR-222-3p induced intestinal oxidative stress and inflammation, possibly by inhibiting the BRG1/Nrf2/HO-1 pathway in IECs, damaged the integrity of the intestinal barrier and induced colon tumorigenesis (**Figure 1**). Furthermore, we found that the downregulation of miR-222-3p relieved oxidative stress in IECs *via* targeting BRG1 to promote the Nrf2/HO-1 signaling pathway, thereby improving colitis and tumorigenesis (**Figure 1**).

First, *in vitro* experiments confirmed that inhibiting miR-222-3p could protect NCM460 colonic cells from DSS-induced oxidative injury (**Figure 2**). Cell viability and caspase-3 experiments showed that treatment with miR-222-3p inhibitor significantly alleviated DSS-induced cell death and apoptosis in NCM460 colonic cells (**Figures 2C, D**). We found that miR-222-3p downregulation reduced oxidative stress and inflammation in DSS-induced NCM460 cells by examining the levels of oxidative stress markers (including ROS, GSH-Px and MDA) and inflammatory factors (including IL-1β and TNF-α) in cells (**Figures 2E-J**). However, the TNF-α results indicated that the difference among samples is small even if there is statistical difference. It may be that the dispersion between samples is small. Therefore, we further verified whether miR-222-3p can regulate TNF-α content in UC and CAC mice *in vivo*. The results showed that the results *in vivo* were consistent with those *in vitro* (**Figure 6Q**) (**Figure 8P**).

In this study, we identified BRG1 as a target gene of miR-222-3p by dual-luciferase assays, and the 3′-UTR of BRG1 has a conserved binding site for miR-222-3p (**Figures 3A, B**). We further showed that miR-222-3p downregulation increased BRG1 expression and promoted the activation of antioxidant Nrf2/HO-1 signaling (**Figures 3C-K**). Previous studies have shown that BRG1 is an important gene that regulates cellular oxidative stress and apoptosis (51, 52), and BRG1 overexpression reverses the lncRNA-TUG1-induced decrease in cell viability and oxidative stress (53). In addition, BRG1 can activate Nrf2/HO-1 signaling, reduce ROS production and MDA levels, and increase the antioxidant enzyme Superoxide Dismutase (SOD), thereby inhibiting oxidative stress and apoptosis (39, 54), while after BRG1 knockout, the expression of Nrf2 and HO-1 significantly decreased, and ROS increased, which in turn aggravated oxidative damage (25, 39, 54, 55). Our results confirmed that miR-222-3p downregulation alleviated DSS-induced apoptosis, oxidative stress and inflammation by promoting BRG1-mediated activation of Nrf2/HO-1 signaling in NCM460 cells *in vitro* (**Figure 4**).

Based on these results, we further evaluated the *in vivo* protective effects of the AAV-miR-222-3p inhibitor using DSS-induced UC mice. After successful AAV-miR-222-3p inhibitor transfection, we extracted mouse IECs. We found that the level of miR-222-3p was dramatically higher in the IECs of DSS-induced UC mice (**Figure 5A**). However, inhibiting miR-222-3p alleviated colon shortening and decreased CMDI and DAI scores in DSS-induced UC mice (**Figures 5B-E**). HE staining showed that colonic damage was significantly improved after UC mice were transfected with the AAV-miR-222-3p inhibitor (**Figures 5F-I**). The reductions in ROS and MDA levels increased GSH-Px levels in IECs further confirmed the enhanced antioxidant effects of the miR-222-3p inhibitor (**Figures 6M–O**), and the miR-222-3p inhibitor markedly reduced the levels of inflammatory markers, including IL-1β and TNF-α, in the supernatants of IECs from DSS-induced UC mice (**Figures 6P, Q**).

Previous studies indicated that BRG1 deletion caused spontaneous colitis in mice, which was accompanied by increased GM-CSF production in intestinal ILC3s. In contrast, the overexpression of BRG1 promoted T-bet-mediated suppression of GM-CSF in ILC3s, thereby ameliorating colitis in Rag1-/-Smarca4ΔILC3 mice (23). Another study showed that BRG1 expression was significantly reduced in the IECs of UC patients, and BRG1 deletion led to excess ROS in mouse IECs, resulting in oxidative stress and apoptosis, which made these animals highly susceptible to DSS-induced colitis (12). Our findings indicated that inhibiting miR-222-3p could activate BRG1 expression in the IECs of UC mice (**Figures 5J-M**) (**Figures 6A-C**).

The Nrf2/HO-1 signaling pathway is an important endogenous antioxidative stress pathway that plays an important role in colitis. By mediating the degradation of Keap1 in the cytoplasm, Nrf2/HO-1 signaling is activated, further significantly increasing the activities of the antioxidant enzymes SOD and GSH-Px, reducing the activities of myeloperoxidase (MPO) and MDA and caspase-3, and inhibiting the levels of the proinflammatory cytokines IL-6, TNF-α and IL-1β in colitis (56, 57). Our findings clearly revealed that the presence of the miR-222-3p inhibitor in IECs attenuated oxidative damage by targeting BRG1 to activate the Nrf2/HO-1 signaling pathway, reduced the protein expression of Keap1, and thereby relieved colonic immune inflammation in DSS-induced UC mice (**Figure 6**) (**Supplementary Figure 3**).

Previous studies have shown that miRNAs play crucial roles in the progression of IBD to CRC (58). Local miRNA expression profile in colon may be involved in the genesis and development of CRC (59). Therefore, after clarifying the pathogenic role of miR-222-3p in the development of UC, we further explored the role of miR-222-3p in CAC mice. Consistent with the increased expression of miR-222-3p in IECs with DSS-induced UC, IECs treated with the miR-222-3p inhibitor were resistant to the development of CAC mice (**Figure 7**), indicating that inhibiting miR-222-3p may regulate CAC development.

Our study identified BRG1 as a target gene of miR-222-3p that regulates IEC function. Inhibiting miR-222-3p can target BRG1 to activate Nrf2/HO-1 signaling in the IECs of CAC mice and reduce Keap1 protein expression (**Figures 7J-M**) (**Figures 8A-K**). BRG1 deletion leads to compensatory regeneration, as well as DNA damage induced by ROS in an inflammatory environment, which promotes the malignant progression of AOM/DSS-induced CRC (12), and Nrf2-knockout mice also exhibit increased sensitivity to CAC (60). This finding was further confirmed by the reduction in CAC in BRG1-overexpressing or Nrf2-overexpressing mice (12, 61). Therefore, previous studies and the present study have confirmed that BRG1 and Nrf2 play important roles in the prevention of inflammation-related colorectal cancer.

Further results showed that inhibiting miR-222-3p reduced the levels of ROS and MDA in IECs, increased the level of GSH-Px, and reduced the levels of the inflammatory factors IL-1β and TNF-α in the supernatants of IECs (**Figures 8L-P**). Previous studies also showed that in the context of oxidative damage in CAC mice, the activities of the antioxidant enzymes GSH-Px and SOD in colon tissue were significantly reduced, and ROS and MDA levels were significantly increased (9, 62). However, increasing the expression of Nrf2 and HO-1 can increase the activity of the antioxidant enzyme SOD, reduce the levels of MDA, inhibit the levels of the inflammatory markers NF-κB, TNF-α, IL-1β and MPO in CAC mice, and have a certain preventive effect on the occurrence of CAC (63, 64). Thus, our data suggest that the presence of a miR-222-3p inhibitor in IECs may protect the gut of CAC mice from oxidative damage and inflammation by targeting BRG1 to activate Nrf2/HO-1 signaling.

In summary, we found that miR-222-3p expression was significantly increased in DSS-induced NCM460 cells and IECs from UC and CAC mice. We identified BRG1 as a target gene of miR-222-3p and that miR-222-3p not only induces intestinal oxidative stress and inflammation, possibly by inhibiting the BRG1/Nrf2/HO-1 pathway in IECs and impairing epithelial integrity but also promotes colon tumorigenesis. However, inhibiting miR-222-3p in IECs attenuates oxidative stress by targeting BRG1 to activate the Nrf2/HO-1 signaling pathway, and thereby alleviating colonic inflammation and tumorigenesis. Because miR-222-3p inhibitors support intestinal inflammation and neoplastic suppression, they could potentially serve as agents for the treatment of patients with UC and CAC and may provide insights into other human diseases related to miR-222-3p.

This study has some limitations. We did not construct miR-222-3p-overexpressing mice. In future studies, we plan to construct miR-222-3p-overexpressing mice to verify whether miR-222-3p overexpression can spontaneously induce colitis and tumorigenesis through oxidative stress. In addition, miR-222-3p inhibitors are not yet in the preclinical development stage. By using miR-222-3p inhibitor, it will be interesting to see if blocking miR-222-3p can prevent/treat colitis and colon tumorigenesis. If successful, a miR-222-3p inhibitor will provide a potential agent for the treatment of patients with IBD or CAC.

## Data availability statement

The original contributions presented in the study are included in the article/**Supplementary Material**. Further inquiries can be directed to the corresponding authors.

## Ethics statement

The animal study was reviewed and approved by Ethics Committee of Yueyang Clinical Medicine School, Shanghai University of Traditional Chinese Medicine (No. YYLAC-2020-094-1, YYLAC-2020-085-1).

## Author contributions

X-JW: Study design, data collection, data interpretation, manuscript preparation. DZ: Study design, manuscript preparation. Y-TY: Study design, manuscript preparation. X-YL: Data collection. H-NL: Data collection. X-PZ: Data collection. J-YL: Data collection. Y-QL: Statistical analysis. LL: Statistical analysis. GY: Data interpretation. JL: Literature search. JH: Literature search. H-GW: Study design, literature search. X-PM: Study design, data interpretation, funds collection. All authors have read and approved the manuscript. All authors contributed to the article and approved the submitted version.
