# Supplementary material for: Suppression of microRNA-222-3p ameliorates ulcerative colitis and colitis-associated colorectal cancer to protect against oxidative stress via targeting BRG1 to activate Nrf2/HO-1 signaling pathway
Source: Front Immunol. 2023 Jan 27;14:1089809. doi: 10.3389/fimmu.2023.1089809 (PMC9911687; doi:10.3389/fimmu.2023.1089809)
Supplement: Supplementary file 1 [file DataSheet_1.docx]

**Supplementary Table**

*Supplementary Table 1 CMDI Scoring*

| Colon macroscopic damage index | | Score | |
| --- | --- | --- | --- |
| Colon adhesion | No adhesions | 0 | |
|  | Minor adhesions | 1 | |
|  | Major adhesions | 2 | |
| Ulcer and inflammation | No ulcer and inflammation | 0 | |
|  | Local congestion without ulcer | 1 | |
|  | 1 ulcer without congestion or bowel wall thickening | 2 | |
|  |  |  | |
|  | 1 ulcer with inflammation | 3 | |
|  | 2 ulcers and inflammation | 4 | |
|  | > 2 ulcers with inflammation or inflammation area > 1 cm | | 5 |
|  | Ulcer and/or inflammation area > 2cm, One more damage, plus 1 | 6~10 | |
|  |  |  | |
|  |  |  | |

*Supplementary Table 2 primer sequences*

| Gene | | PrimerF | PrimerR |
| --- | --- | --- | --- |
| has-miR-222-3p | 5' AACAAGAGCTACATCTGGCTACTG3' | - |  |
| has-BRG1 | | 5' GACCAGCACTCCCAAGGTTAC3' | 5' CTGGCCCGGAAGACATCTG3' |
| has-Nrf2  has-HO-1 | | 5' TTCCCGGTCACATCGAGAG3'  5' AAGACTGCGTTCCTGCTCAAC3 | 5' TCCTGTTGCATACCGTCTAAATC 3'  5' AAAGCCCTACAGCAACTGTCG3' |
| has-GAPDH  U6 | | 5' AAAATCAAGTGGGGCGATGC3'  5' CCTGCTTCGGCAGCACA3' | 5' TGGTTCACACCCATGACGAA3'  - |
| mmu-miR-222-3p | | 5' AACACGCAGCTACATCTGGCTA3' | - |
| mmu-BRG1 | | 5' GGTTCTGCCCACAGCATGAT3' | 5' GGACTCCATAGGCTTGTGCAT3' |
| mmu-Nrf2 | | 5' CTTTAGTCAGCGACAGAAGGAC3' | 5' AGGCATCTTGTTTGGGAATGTG3' |
| mmu-HO-1 | | 5' CAGAACCCAGTCTATGCCCC3 | 5' GTGAGGCCCATACCAGAAGG3' |
| mmu-GAPDH | | 5' AGGTCGGTGTGAACGGATTTG3' | 5' TGTAGACCATGTAGTTGAGGTCA3' |

Supplementary Table 2 has: human gene primer sequences, mmu: mouse gene primer sequences.

**Supplementary *Figure***

*Supplementary Figure 1* Fluorescence identification of AAV transfection in UC


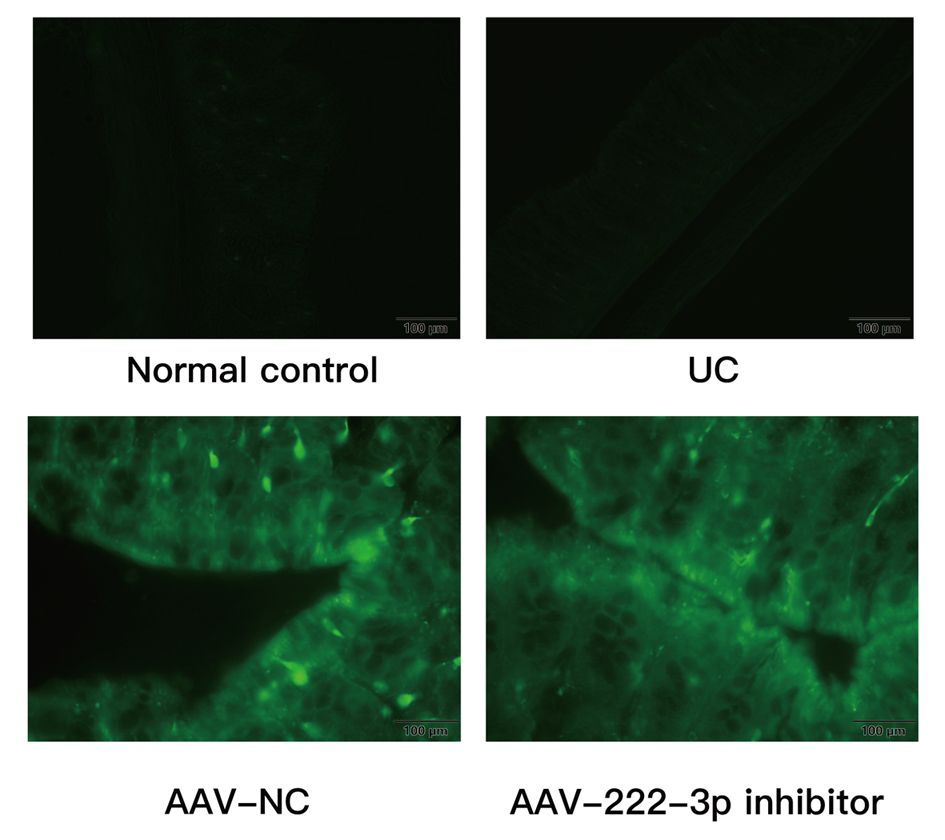


Supplementary Figure 1 AAV9-GFP fluorescence intensity of each group. Normal control：Normal control group；UC：UC group；AAV-NC：UC+ AAV-negative control group；AAV-222-3p inhibitor：UC+ miR-222-3p inhibitor group. Scale bar: 100 *µ*m.

*Supplementary Figure 2* Fluorescence identification IECs


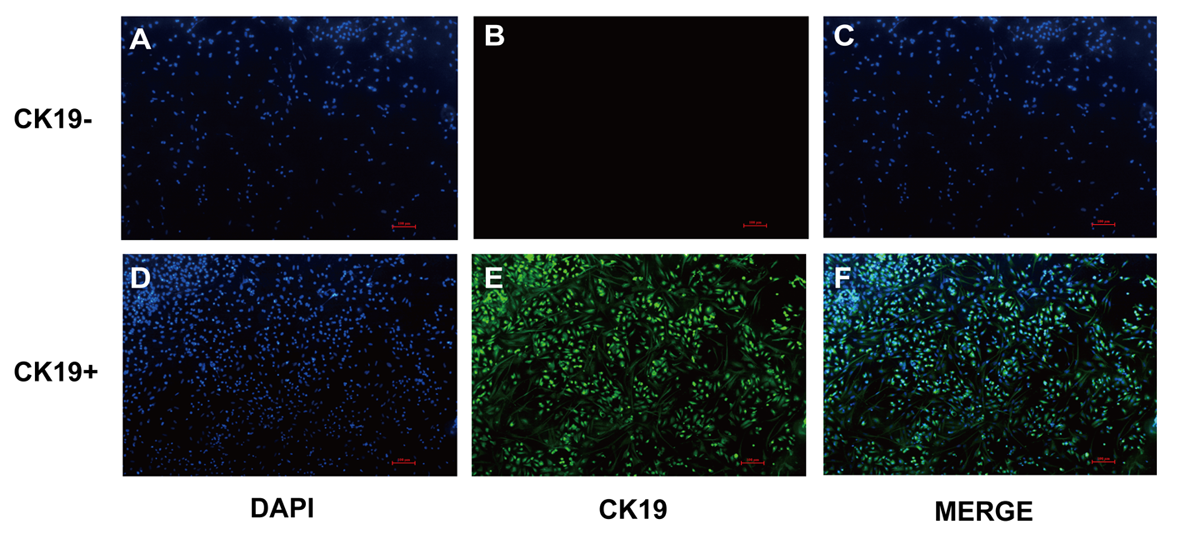


Supplementary Figure 2 Single immunofluorescent staining for cytokeratin 19 (CK 19+) or without CK 19 (CK 19-) were performed in the IECs. Nuclei was stained with DAPI in blue. CK 19 was stained in green. Scale bar: 100 *µ*m.

*Supplementary Figure 3* Relative caspase-3 protein expression in the IECs from DSS-induced mice.


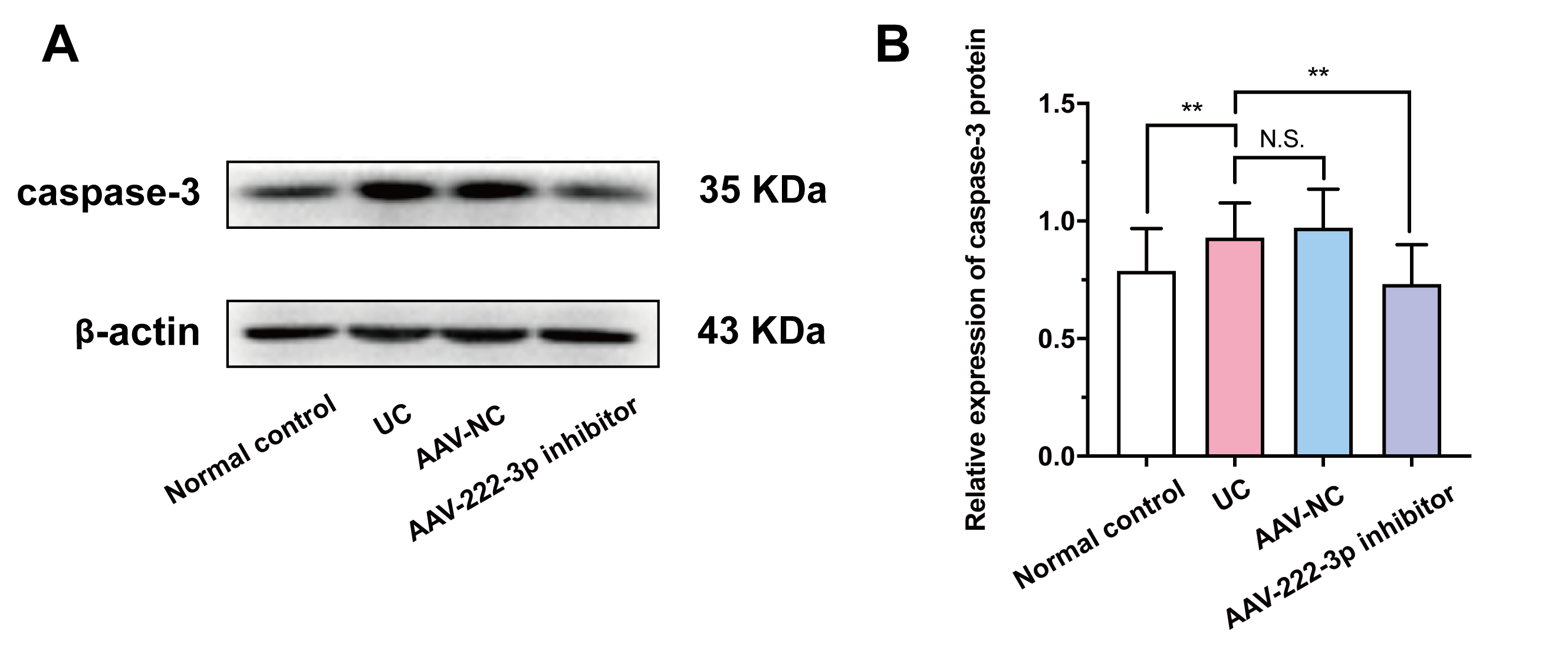


Supplementary Figure 3 Relative caspase-3 protein expression was determined by Western blot in the IECs from DSS-induced mice. Data are presented as the mean ± SD (n = 8). ^*^*P* < 0.05, ^**^*P* < 0.01, ^***^*P*<0.001. Normal control：Normal control group；UC：UC group；AAV-NC：UC+ AAV-negative control group；AAV-222-3p inhibitor：UC+ miR-222-3p inhibitor group.

*Supplementary Figure 4* Fluorescence identification of AAV transfection in CAC


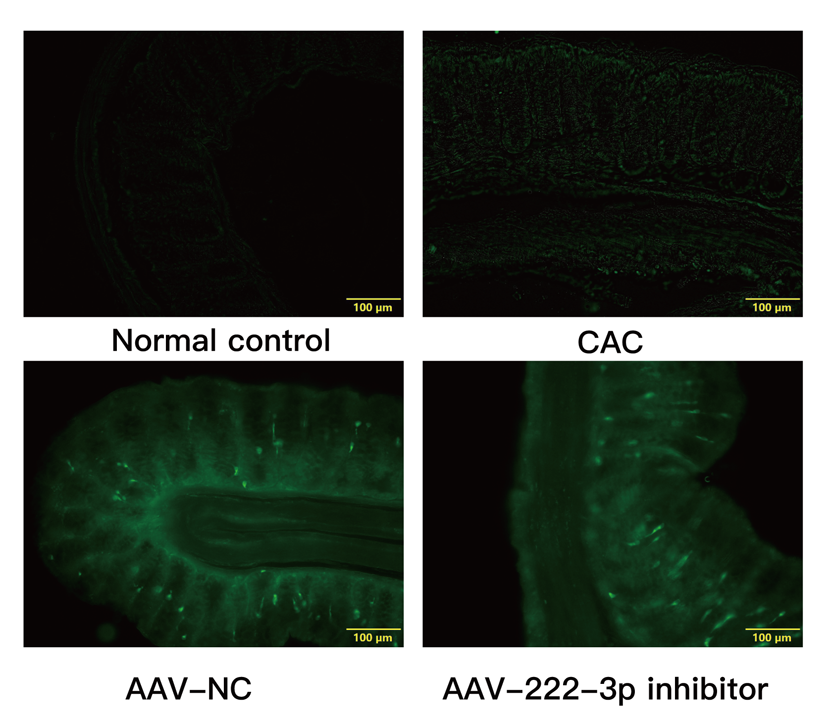


Supplementary Figure 4 AAV9-GFP fluorescence intensity of each group. Normal control：Normal control group；CAC：CAC group；AAV-NC：CAC+ AAV-negative control group；AAV-222-3p inhibitor：CAC+ miR-222-3p inhibitor group. Scale bar: 100 *µ*m.
